# Histologic and ultrastructural study of intracranial Gaucheroma causing deafness in a patient with Gaucher disease type 3: Effects of substrate reduction therapy

**DOI:** 10.1016/j.ymgmr.2024.101106

**Published:** 2024-06-14

**Authors:** Shoji Yano, Rachel McGowan, Mikako Warren

**Affiliations:** a Genetics Division, Pediatrics, Los Angeles General Hospital, University of Southern California, Los Angeles, CA, USA; b Pathology and Laboratory Medicine, Children's Hospital Los Angeles, University of Southern California, Los Angeles, CA, USA

**Keywords:** Histopathological study, Enzyme replacement therapy (ERT), Gaucher disease, Hearing loss, Gaucheroma, Substrate reduction therapy (SRT)

## Abstract

Hearing loss is frequently associated with Gaucher disease (GD). Gaucher cells are enlarged reticuloendothelial cells containing glucocerebroside in the lysosomes due to deficiency of the glucocerebrosidase. Gaucheromas consist of accumulated Gaucher cells. Gaucher cells accumulate in variable tissues including the liver, spleen, bone marrow, and the middle ear and the mastoid causing conductive hearing loss. Neurons and astrocytes in the central nervous system are affected in neuronopathic GD leading to sensorineural hearing loss. Gaucheromas can develop even in patients treated with enzyme replacement therapy (ERT). We report a 19-year-old female patient with GD type 3 who developed profound bilateral hearing loss associated with intracranial Gaucheroma. Combination therapy of ERT with imiglucerase and substrate reduction therapy (SRT) with eliglustat significantly decreased the size of Gaucher cells and cleared the characteristic microtubular structures in the lysosomes in Gaucher cells. Early implementation of SRT may prevent at least conductive hearing impairment in GD although it may not prevent sensorineural hearing loss due to inner hair cell dysfunction which is also known to be associated with neuronopathic GD.

## 1 Introduction

Gaucher disease (GD) is an autosomal recessive disorder due to mutations in *GBA1* encoding lysosomal glucocerebrosidase. GD is the most common lysosomal storage disorder and three major clinical types are known: Type 1 non-neuronopathic, Type 2 acute/infantile neuronopathic, and Type 3 subacute/juvenile neuronopathic. Defective lysosomal glucocerebrosidase causes the accumulation of glucocerebroside in the lysosomes of cells derived from the monocyte and macrophage lineage, which are known as Gaucher cells (GCs). Neurons and astrocytes in the central nervous system are affected in neuronopathic GD. GCs infiltrate the various organs and accumulate forming pseudotumor, i.e., Gaucheromas. Gaucheromas are rare complications in GD and enzyme replacement therapy does not seem to prevent them from developing. Although the mesenteric, mediastinal, and cervical lymph nodes are often the primary sites, Gaucheromas infiltrating the mastoid and the middle ear resulting in conductive hearing loss in Gaucher type I disease have been reported [1,2]. Sensorineural hearing loss has been reported in patients with neuronopathic GD [3,4]. Sounds are perceived by sensory neurons, called hair cells, in the cochlea of the inner ear. The mammalian target of rapamycin complex 1 (mTORC1) hyperactivity is reported in GD [5]. mTORC1 overactivation is thought to be involved in inner hair cell damage, which may also contribute to sensorineural hearing loss in neuronopathic GD. We report a case of GD type 3 who started enzyme replacement therapy (ERT) in her infancy, developed profound mixed hearing loss at age 4 y, and was diagnosed with intracranial Gaucheroma at age 16 years.

## 2 Case presentation

**Fig. 1:**
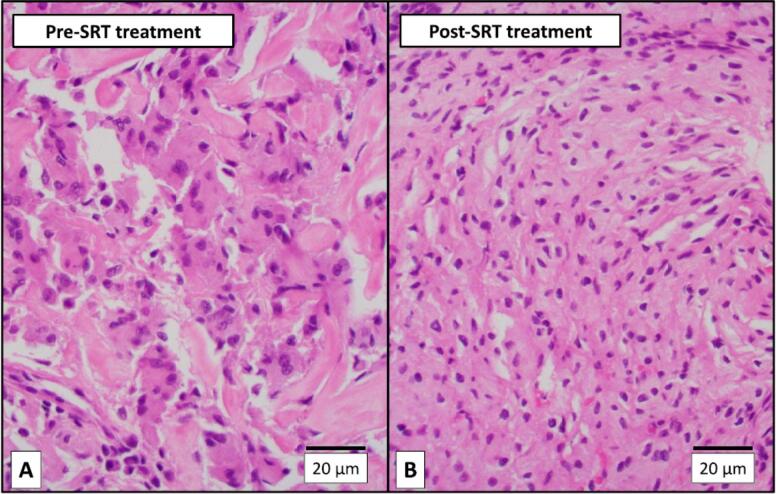
H&E (200X) microphotographs of the pre-SRT treatment Gaucheroma (A) and the post-treatment tissue from the same site (B). The Gaucheroma comprised numerous large histiocytic cells with a characteristic “wrinkled tissue paper” appearance (Gaucher cells). The post-treatment tissue showed histiocytes significantly smaller than Gaucher cells. The histiocytes lacked a “wrinkled tissue paper” appearance and were no longer “Gaucher cells.”

**Fig. 2:**
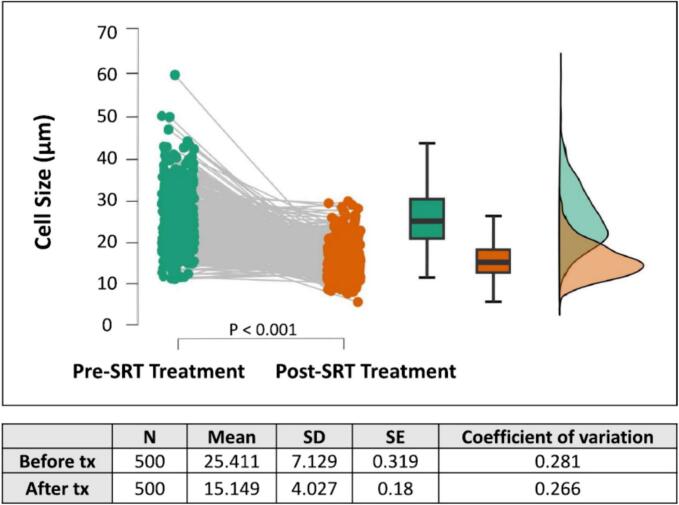
The graph shows that the post-treatment histiocytic cell sizes were significantly smaller than those in the pre-SRT treatment specimen. The histiocytic size distribution also became narrower after the treatment.

**Fig. 3:**
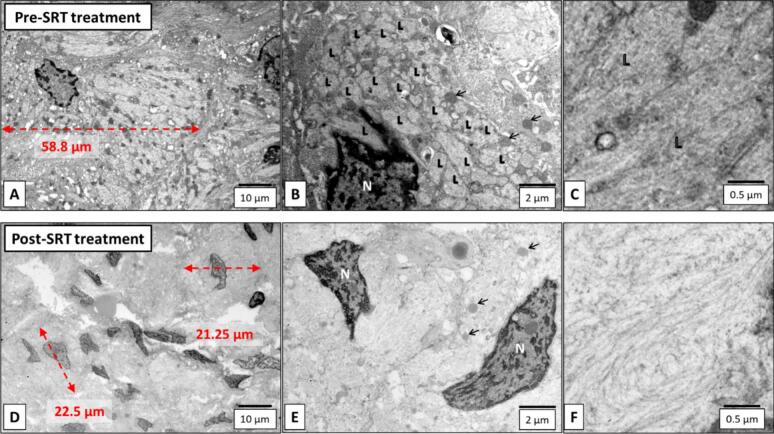
Electron micrographs of the pre-SRT specimen (Gaucheroma, A-C) and post-SRT treatment specimen (D—F). Fig. A and B demonstrates that the pre-treatment specimen contained large-sized histiocytes with abundant enlarged lysosomes (L: lysosomes; N: nuclei; black arrows: mitochondria). Fig. C shows high-magnification view of the lysosomes that are filled with microtubular structures in the cell measuring 50–70 μm consistent with Gaucher cells. In the posts-SRT specimen, the histiocytes are smaller than those in the pre-treatment specimen (D), and have an “empty” appearance. It is difficult to find lysosomes (E), and microtubular structures became inconspicuous and only cytoskeleton structures are visible (F).

A 19-year-old female with type 3 GD diagnosed at birth based on the *GBA1* genotype (homozygous L444P) was treated using ERT with imiglucerase (120 IU/kg/2 weeks) from age 2 months. She developed profound mixed hearing loss at age 4 y. Surgical procedure to place a cochlear implant at age 16 y was aborted when a mass of abnormal tissue extending into the middle ear space and mastoid was found, later histopathologically diagnosed with GCs. After 51 months of substrate reduction therapy (SRT) with eliglustat in addition to ERT, a cochlear implant was placed. Light and electron microscopic findings were compared between the specimens from the pre- and the post-SRT. The mean size of GCs significantly decreased after SRT (pre: 25.4 ± 7.1, post: 15.1 ± 4.0 mm, *p* < 0.01, *n* = 500, Fig. 1, Fig. 2). Electron micrographs showed the normalized size of lysosomes and cleared intra-lysosomal microtubular structures in the post-SRT specimen (Fig. 3).

## 3 Discussion

GC infiltration in the organs involved in hearing has been reported in patients with type I and type III GD who developed deafness [1,2,4]. Although ERT with imiglucerase alone could not prevent intracranial GC infiltration forming Gaucheroma, supplementation of SRT with eliglustat decreased the size of GCs in the presented case (Fig. 1, Fig. 2, Fig. 3). Since intracranial Gaucheroma is outside of the blood brain barrier, early implementation of SRT may prevent at least conductive hearing impairment. To the best of our knowledge, histologic and ultrastructural studies of Gaucheromas showing significant decrease in size in GCs and clearance of the accumulated abnormal lysosomal microtubular structure after initiation of SRT have never been reported.

An inner hair cell dysfunction due to glucocerebroside toxicity is also thought to be involved to cause sensorineural deafness [5,6]. Accumulation of glucocerebroside leads to increase mTORC1 activity which results in increased transcription factor EB (TFEB) phosphorylation, leading to proteasomal degradation of TFEB and subsequent downregulation of lysosomal functions in the hair cells.  Drug delivery to the inner ear hair cell requires crossing the blood labyrinth barrier (BLB). Since eliglustat crosses the blood-brain barrier [7], it may cross the BLB based on the similarity in their structures and protein composition including the drug efflux transporter ABCB1 [8,9]. BLB functions as a physical barrier as well as a biochemical barrier with efflux pump systems including the ABC transporter (ATP-binding cassette transporter protein 1 encoded by the ABCB1 gene) which is known immediately to transport eliglustat back out of the central nervous system [7].  To prevent sensorineural hearing impairment by preserving the hair cells in neuronopathic GD, there might be beneficial effects in use of mTOR inhibitors as well as P-glycoprotein inhibitors (ABCB1 inhibitors) along with use of SRT.

## 4 Conclusion

We reported, for the first time, a case of Gaucher disease Type 3 treated with ERT with imiglucerase since infancy, developed bilateral profound mixed hearing loss associated with intracranial Gaucheromas, which significantly reduced in the size of GCs and cleared abnormally accumulated microtubular structures in the lysosomes in the GCs after implementation of SRT with eliglustat. ERT alone does not seem to have significant beneficial effects on GCs in Gaucheromas, but SRT with eliglustat clearly showed significant beneficial effects. Further analyses with more cases are needed to evaluate indication of early implementation of SRT with eliglustat as well as indication of mTOR inhibitors and ABCB1 transporter inhibitors to prevent hearing impairment in GD.

## Ethics statement

This article does not contain any experimental studies with human or animal subjects performed by any of the authors. This article reported the observational findings seen in a patient who received the standard management. All procedures followed were in accordance with the ethical standards of the responsible committee on human experimentation (institutional and national) and with the Helsinki Declaration of 1975, as revised in 2000 (5). This single case study is IRB exempt at our institution.

## Informed consent

Informed consent was obtained. Proof that informed consent was obtained is available upon request.

## CRediT authorship contribution statement

**Shoji Yano:** Conceptualization, Project administration, Writing – original draft, Writing – review & editing, Supervision. **Rachel McGowan:** Writing – review & editing. **Mikako Warren:** Formal analysis, Investigation, Validation.

## Declaration of competing interest

All authors declare no conflicts of interest.

Funding

None.

## Data Availability

No data was used for the research described in the article.
